# The Joint Contribution of Host Genetics and Probiotics to Pig Growth Performance

**DOI:** 10.3390/microorganisms13020358

**Published:** 2025-02-07

**Authors:** Jinyi Han, Mingyu Wang, Shenping Zhou, Zhenyu Wang, Dongdong Duan, Mengyu Li, Xiuling Li, Wenshui Xin, Xinjian Li

**Affiliations:** 1 College of Animal Science and Technology, Henan Agricultural University, Zhengzhou 450002, China; 2 Sanya Institute, Hainan Academy of Agricultural Sciences, Sanya 572000, China

**Keywords:** performance of growth, pigs, gut microbiota, BMI, MGWASs

## Abstract

Intestinal probiotics significantly regulate the growth performance of their host, with their composition being influenced by various factors. While many studies have explored how gut microbiota composition affects growth traits such as body weight and BMI, the research on probiotics influenced by host genetic factors, and their subsequent impact on host growth performance, remains limited. To address this research gap, we collected fecal and tissue samples, as well as phenotypic data, from 193 Yunong black pigs at 280 days of age. We then sequenced and genotyped all 193 subjects using the 50K SNP BeadChip, yielding a comprehensive dataset for genetic and microbiome analyses. We then employed microbiome-wide association studies (MWAS), a meta-analysis, and microbiome-wide genetic association studies (MGWASs) to examine the relationship between host genetics, gut microbiota, and growth performance. Four key microbial taxa, namely *Coprococcus*, *Blautia*, *Ruminococcaceae*, and *RF16*, were identified as being significantly associated with body weight and BMI. The MGWAS analysis revealed that both *Coprococcus* and *Ruminococcaceae* were significantly associated with host genomic variations. A total of four important single nucleotide polymorphisms (SNPs) were mapped to two chromosomal regions, corresponding to three candidate genes. Among them, the candidate genes *INPP4B*, *SCOC*, and *PABPC4L* were identified as being related to the abundance of key microbes. This study provides new insights into the joint contributions of host genetics and probiotics to host growth traits, offering theoretical guidance and data support for the development of efficient and targeted breeding strategies.

## 1. Introduction

The growth performance of pigs, a key feature in animal husbandry, is closely tied to the economic profitability of the pig industry. Indicators such as body weight and BMI reflect the pigs’ nutritional efficiency and overall health. Research has demonstrated that pig growth performance is influenced by a variety of factors, including nutrition, genetics, environment, and health [1,2]. Notably, gut probiotics play a crucial role in regulating this performance. Differences in the gut microbiota composition can affect the host’s nutrient absorption capacity, thereby influencing its growth performance.

Gut microbiota play a significant role in both the health and production efficiency of pigs. A large number of microorganisms reside in the animal’s gut, where they are directly involved in the host’s absorption of carbohydrates, fats, and proteins. Studies have shown that probiotics can be added to animal feed as growth promoters, thereby influencing food intake and weight gain in animals [3]. Furthermore, research has found that high abundances of *Escherichia* and *Lactobacillus* in the ileum contribute to the degradation of glucose and oligosaccharides [4,5]. Actinobacteria in the cecum promote polysaccharide fermentation [6], while *Lactobacillus* and *Streptococcus* in the colon play significant roles in lactate production [7]. These studies collectively demonstrate that the gut microbiota can influence nutrient absorption in the host through the metabolism of dietary components, thus affecting the host’s growth performance.

On the other hand, research also suggests that gut microbiota can impact the host’s growth performance by improving host health. Studies have shown that microbial adhesion to the intestinal mucosa forms a biofilm barrier, where the S-layer protein of *Lactobacillus* can specifically adhere to host intestinal epithelial cells [8], protecting the gut barrier and preventing the invasion and colonization of pathogenic bacteria. In addition, the surface layer protein A of *Lactobacillus acidophilus* can bind to colorectal cancer-associated cells, inducing the expression of tight junction protein ZO-1, which strengthens tight junctions and reduces cellular permeability [9,10]. A study by Liang et al. demonstrated that *Clostridium butyricum* effectively reduced weaning stress in piglets, decreasing their diarrhea rate and improving their growth performance [11]. These studies highlight the critical role of gut microbiota in regulating host growth performance.

Although numerous studies have elucidated the relationship between gut microbiota and host growth performance, the interaction between host genetics and gut microbiota in influencing host growth remains unclear. Recent studies have suggested that host genetic factors can lead to changes in the gut microbiome of chickens with different growth performance [12,13]. Zhao et al. used metagenomic sequencing to analyze the gut microbiota composition in fecal samples from 60 high-weight (HW) and low-weight (LW) adult chickens of two strains, raised in identical environmental conditions. Their results showed significant differences in 29 bacterial species between the two strains, providing preliminary evidence that host genetics may influence gut microbiome composition [14]. In cattle, Fan et al. studied the effect of host genetics on the gut microbiota structure across three different growth stages in hybrid beef cattle raised under varying environmental and dietary conditions [15]. Their findings indicated that the gut microbiota was significantly influenced by the host genetic background throughout the animal’s life, regardless of the growth stage. However, the potential host genotypes related to pig gut microbiota have yet to be fully established. Recently, Yang et al. reported a significant association between the host ABO genotype and the abundance of gut microbiota in pigs, suggesting that host genetic variation may affect the composition of the pig gut microbiota [16].

In this study, we measured and analyzed the body weight and BMI of 193 280-day-old Yunong black pigs, and selected individuals with extreme phenotypic differences for a gut microbiome diversity analysis. The goal was to identify candidate microbial communities significantly associated with pig growth performance. Subsequently, all 193 individuals underwent 50K genotyping chip sequencing and 16S rRNA sequencing, followed by an MGWAS analysis. This analysis aimed to assess the impact of host genetics on the abundance of key microbes, with the objective of identifying microbial species regulated by host genetics that are associated with growth performance, as well as potential genetic markers linked to these associations. This study aims to elucidate the interaction between microbiota and host genetics, evaluate their combined influence on pig growth performance, and provide theoretical insights for the development of efficient and targeted genetic breeding strategies.

## 2. Methods

All of the experiments involving animals were carried out in accordance with the guidelines for the care and use of experimental animals established by the Ministry of Science and Technology of the People’s Republic of China (Approval Number DWLL20211193). The animal study was reviewed and approved by the Henan Agricultural University Animal Care and Use Committee. In addition, all experiments were conducted in accordance with the relevant approved guidelines and regulations during sampling and sample conservation.

### 2.1. Animals and Phenotyping

This study involved 193 Yunong black sows, each 180 days old, selected for 16S rRNA sequencing, genome sequencing, and phenotype evaluation. All animals were housed and fed using Osborne automated feeding stations (Osborne Industries Inc., Osborne, KS, USA). All sows used in the experiment were housed in different pens within the same pig house, which was equipped with temperature and humidity control facilities such as water curtains. The temperature in the pig house was maintained at 20–23 °C, and the humidity was kept between 65% and 80%. All experimental animals were fed the same diet composed of corn and soybeans (Table 1). All the measured data were corrected and analyzed by IBM SPSS Statistics 22.0.

Body weight and body length were measured in a cohort of 195 age-matched Yunong black pigs. The BMIs of the pigs are calculated as follows [17]:

$$BMI=\frac{Body\;weight\;(kg)}{{Body\;length\;(m)}^{2}}$$

Arranged in ascending order of BMI or BW, five gradients were established. These gradients were designed to observe the dynamic changes in microbial structure. Finally, 193 Yunong black pigs were selected for 16S rRNA sequencing and genomic sequencing to be used in the MGWAS analysis.

The premix provides the following per kilogram of feed: vitamin A 10,000 IU; vitamin D3 1800 IU; vitamin E 100 IU; vitamin K3 4.5 mg; vitamin B1 2.0 mg; riboflavin 6.0 mg; vitamin B6 7.0 mg; vitamin B12 0.05 mg; niacin 30 mg; pantothenic acid 35 mg; folic acid 3.5 mg; biotin 0.5 mg; choline chloride 500 mg; iron 80 mg; copper 20 mg; zinc 100 mg; manganese 25 mg; iodine 0.14 mg; and selenium 0.15 mg.

### 2.2. Sample Collection

Use a new nitrile examination glove to palpate the rectum and collect the rectal contents from each animal. The samples were then placed into cryovials and immediately placed in liquid nitrogen. All samples were immediately shipped to the laboratory and stored at −80 °C until further analysis.

### 2.3. DNA Extraction and Polymerase Chain Reaction (PCR) Amplification

Microbial DNA was extracted using the OMEGA Soil DNA Kit (OMEGA Bio-Tek, Norcross, GA, USA) and subsequently stored at −20 °C prior to analysis. The V3–V4 region of the 16S rRNA gene was amplified from these DNA extracts using the forward primer (5′-ACTCCTACGGGAGGCAGCA-3′), the reverse primer (5′-GGACTACHVGGGTWTCTAAT-3′) and the final 5′-Illumina adapter. The PCR cycling conditions consisted of an initial denaturation step at 95 °C for 5 min, followed by denaturation at 25 °C.

### 2.4. 16S rRNA Gene Sequence Assembly and Clustering

16S rRNA sequence data were processed using the QIIME2 2019.4 platform [18]. The sequences were filtered, denoised, and concatenated using the DADA2 plug-in to remove the highest quality chimeras. The ASVs were aligned using the MAFFT algorithm. We then used the Greengenes database (http://greengenes.lbl.gov, accessed on 5 October 2024) to classify the sequences using the classifier Sklearn algorithm with QIIME2 default parameters.

### 2.5. Bioinformatics and Statistical Analysis

Statistical comparisons of taxonomic abundance at the phylum and genus levels were performed between groups. Different methods were used to analyze and visualize the alpha and beta diversity indices at the ASV level. Observed species and alpha diversity metrics such as the Shannon diversity index were calculated based on the ASV table in QIIME2 and visualized as boxplots.

### 2.6. Genotype Data Acquisition and Quality Control

Genomic DNA was extracted from 461 black pig ear tissues using the phenol-chloroform method. DNA quality was determined by UV spectrophotometry and gel electrophoresis. The DNA samples were then genotyped using the KPS Pig Breeding Chip 50K(Beijing Compass Agritechnology Co., Ltd., Beijing, China). PLINK v1.9 was used for quality control to screen for minor alleles with calling rate < 0.90, frequency < 0.05, and significant SNPs deviating from Hardy–Weinberg.

### 2.7. MWAS Analysis

To identify microbes that significantly influence BMI or body weight (BW), an analysis of variance (ANOVA) was performed to evaluate the differences in microbial composition between pigs with a high body weight (HWB, *n* = 40), low body weight (LWB, *n* = 40), high BMI (HBMI, *n* = 40), and low BMI (LBMI, *n* = 40). Furthermore, the Wilcoxon rank-sum test was conducted to evaluate the relative abundance of each taxon between the highest (E groups; *n* = 40) and lowest (A groups; *n* = 40) BMI or BW pigs. A microorganism was deemed significant if the adjusted *p* values from both the ANOVA and the Wilcoxon rank-sum test in the two-part model association analysis were less than 0.05. The Spearman and Pearson correlations between groups were calculated using the Psych package in R (v4.4.1), and the *p* values were adjusted using the BH method. A correlation was considered significant if the adjusted *p* value was <0.05.

### 2.8. GWAS Analysis

In this study, we performed a GWAS analysis on the genetic and phenotypic data from 461 sows using the R rMVP package (v4.4.1). The statistical model is summarized as follows:

y=Qα+Xβ+Zu+g+e

where y  represents the observed phenotype vector (relative abundance of the microbiota); and Q denotes the covariate matrix, which consists of the first five host genetic principal components to correct for the influence of the host’s genetic structure on the trait. α represents the effect coefficient of the host genetic principal components on the trait, indicating the linear relationship between each principal component and the trait. X is the fixed effect matrix, used to account for environmental factors affecting the trait, as the data comes from different pig farms. β denotes the fixed effect coefficient; and Z is the random effect matrix, used to correct for the influence of potential genetic correlations on the trait. u represents the vector of random effects, indicating the individual-specific effects for each sample; g represents the individual random effect; and e denotes the residual vector.

Therefore, we employed the false discovery rate (FDR) to establish the significance thresholds for the GWAS analysis. The FDR was set at 0.01, and the threshold *p* value was calculated using the following formula:

$$P=\frac{FDR\times n}{m}$$

where n represents the number of SNPs with *p* < 0.01 in the results, and m is the total number of SNPs analyzed [19].

A linkage disequilibrium analysis was performed using PLINK v1.9.0, and LD blocks were generated using HAPLOVIEW v4.2 under the default parameters. According to the SSCROFA 11.1 reference genome, the genes closest to significant SNPs were identified as candidate genes.

Using the ENSEMBL Sscrofa 11.1 database, the genes closest to important sites within 0.5 Mb upstream or downstream were identified. These genes were then imported into KOBAS (http://bioinfo.org/kobas, accessed on 25 November 2024) for Gene Ontology (GO) and Kyoto Encyclopedia of Genes and Genomes (KEGG) enrichment analysis.

## 3. Results

### 3.1. Gut Microbiome Diversity Analysis in Yunong Black Pigs

The gut microbiota plays a crucial role in the host’s digestive and absorptive functions. To assess its impact on host growth performance, we performed 16S rRNA sequencing on the rectal contents of pigs. After quality filtering, a total of 17,034,696 sequence reads were obtained, with an average of 87,357 reads per sample (ranging from 48,937 to 177,548). Taxonomically, we identified 40 phyla, 124 classes, 241 orders, 419 families, and 938 genera across all pig rectal samples.

**Figure 1:**
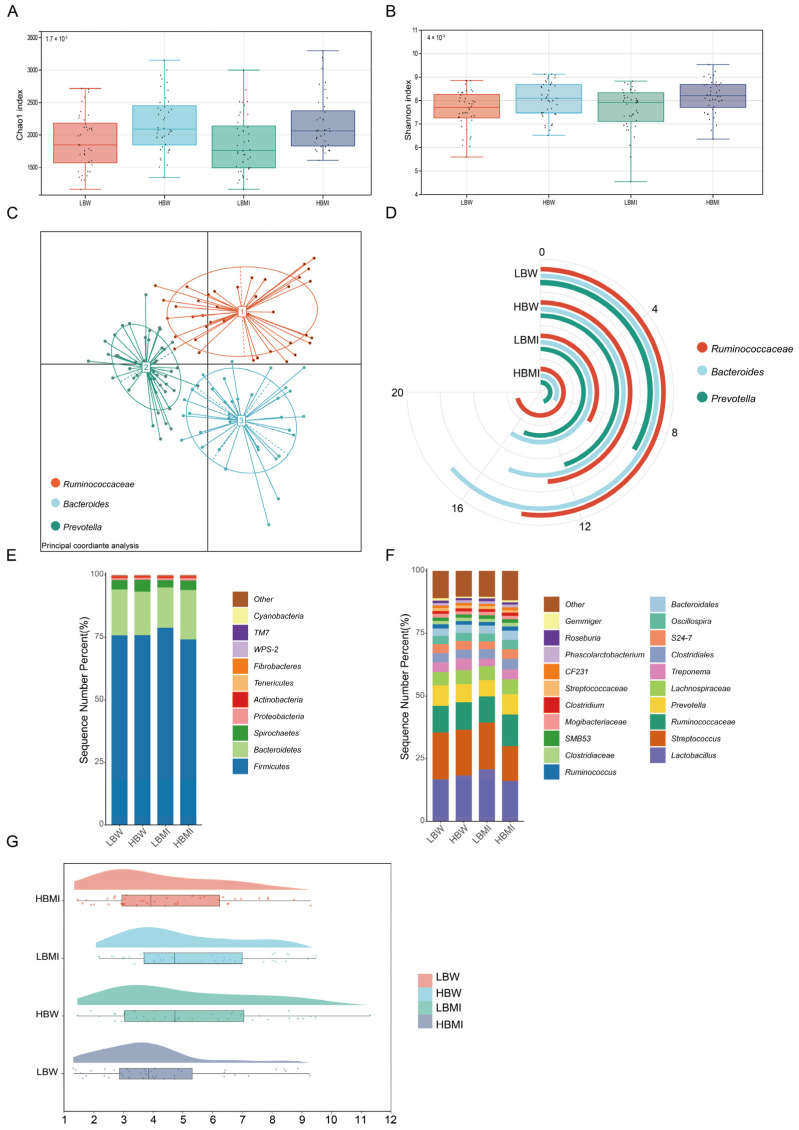
Gut microbiome diversity analysis. (**A**,**B**) Alpha diversity analysis of the gut microbiome in Yunong black pigs with different growth performances. (**C**,**D**) Analysis of enterotype composition in Yunong black pig population. (**E**) Stacked bar chart of microbiota abundance at the phylum level. (**F**) Stacked bar chart of microbiota abundance at the genus level. (**G**) Dynamic changes in the F/B ratio across different ADG groups. HWB—pigs with the highest BW. LWB—pigs with the lowest BW. HBMI—pigs with the highest BMI. LBMI—pigs with the lowest BMI.

Alpha diversity analysis is a widely used method in gut microbiome studies, providing insights into the complexity and variability of microbial communities. This approach offers researchers a comprehensive understanding of the overall gut microbiota composition. In this study, we analyzed the alpha diversity of pigs with different BW and BMI phenotypes. Our results showed that both the Chao1 and Shannon indices were significantly higher in the HBW group compared to the LBW group (*p* < 0.05), and similarly, the HBMI group exhibited significantly higher Chao1 and Shannon indices than the LBMI group (*p* < 0.01). These findings suggest that the HBW and HBMI groups have a greater diversity of microbial species, with higher microbial richness, which contributes to a healthier and more stable gut microbiota structure, thus facilitating better nutrient digestion and absorption (Figure 1A,B).

**Figure 2:**
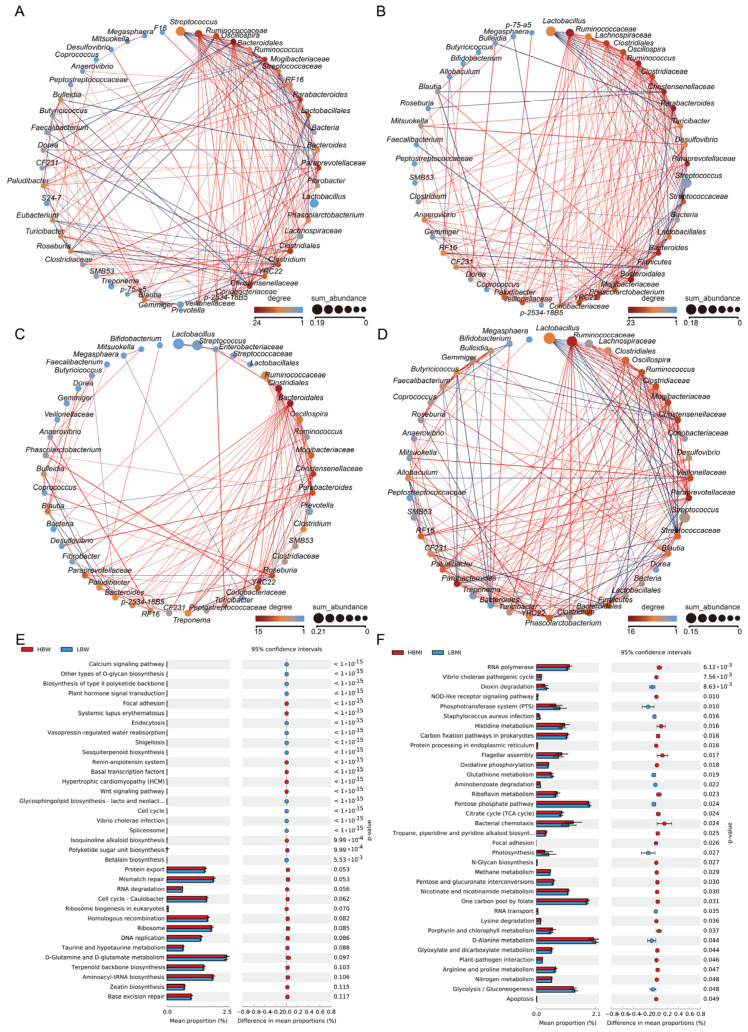
Gut microbiota interaction networks and differential functional and pathway enrichment analysis. (**A**) Gut microbiota interaction network in the LBW group. (**B**) Gut microbiota interaction network in the HBW group. (**C**) Gut microbiota interaction network in the LBMI group. (**D**) Gut microbiota interaction network in the HBMI group. (**E**,**F**) Differential GO and KEGG enrichment analysis of gut microbiota functions and pathways. Note: in the interaction networks, the node color represents the core level of the microbiota, with a darker red indicating a higher core level; the node size reflects the relative abundance, with larger nodes indicating a higher abundance; the color of the connections between nodes indicates the correlation, with red indicating a positive correlation and blue indicating a negative correlation. HWB—pigs with the highest BW. LWB—pigs with the lowest BW. HBMI—pigs with the highest BMI. LBMI—pigs with the lowest BMI.

In the gut type analysis, we observed that the Yunong black pig population is composed of three distinct gut types, with different proportions of each type observed in subgroups displaying varying growth performances. This trend suggests that the nutritional absorption environment in Yunong black pigs is generally healthy, although the functionality of their gut microbiomes shows some variability (Figure 1C,D). Building on these preliminary results, we further explored the composition of the gut microbiota in Yunong black pigs and its relationship with different BW and BMI phenotypes. At the phylum level, the microbial communities in all groups were predominantly composed of *Firmicutes*, *Bacteroidetes*, *Spirochaetes*, and *Proteobacteria*, with no significant differences observed between the groups. At the genus level, *Lactobacillus*, *Treponema*, *Prevotella*, *SMB53*, and *Oscillospira* were the dominant genera identified in the rectal content samples, collectively accounting for approximately 34.91% of the total microbial composition (Figure 2E,F).

In a further analysis, we assessed the ratio of *Firmicutes* to *Bacteroidetes* (F/B ratio) in the gut microbiota of pigs with different BW and BMI phenotypes. We found that the F/B ratio in the HBW group was significantly higher than in the LBW group (*p* < 0.05), indicating a greater proportion of Firmicutes in the HBW group, which could be associated with obesity and metabolic syndrome. In contrast, the F/B ratio in the HBMI group was significantly lower than in the LBMI group (*p* < 0.05), suggesting improved nutrient absorption and reduced energy expenditure, which is advantageous for fattening pigs. Overall, these findings provide strong evidence that the gut microbiota plays a significant role in influencing the growth performance of the host.

### 3.2. Construction of Intestinal Microecological Network and Enrichment of Differential Functions

**Figure 3:**
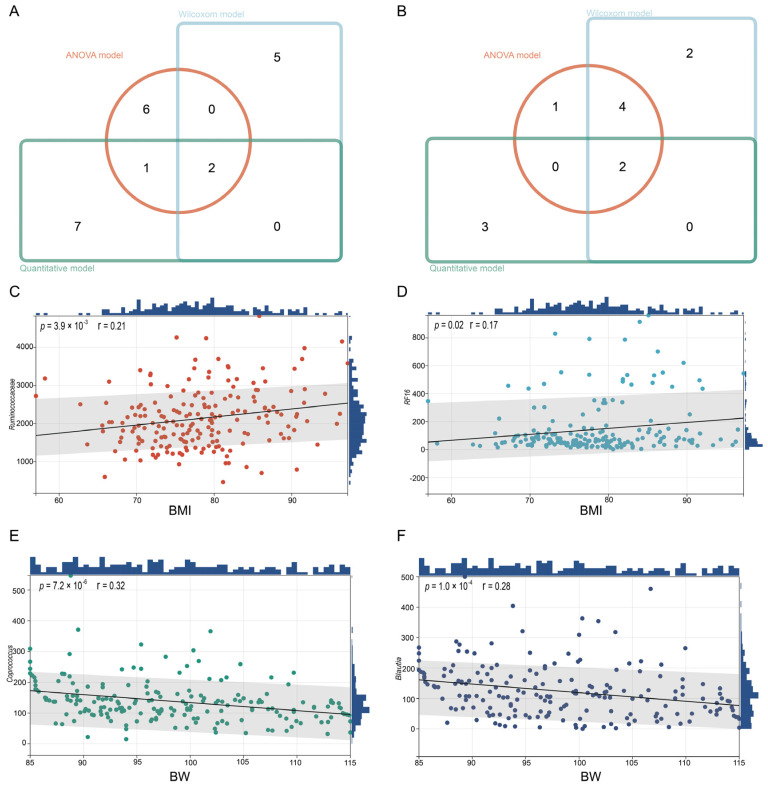
MWAS analysis and meta-analysis results. (**A**,**B**) Results of MWAS analysis, meta-analysis, and Venn diagram; and (**C**–**F**) relationship between key microbiota and phenotype.

To comprehensively and clearly assess the gut microbiome structure of Yunong black pigs with different growth performance phenotypes, we established interaction networks for the top 50 most abundant microbial communities in the intestines of pigs from the HBW, LBW, HBMI, and LBMI groups. The results are shown in Figure 3A–D. The microbial interaction network of the LBW group was relatively simple, while that of the HBW group was more complex. Specifically, we identified core microbes (degree ≥ 20) that regulate the abundance of other microbial communities. In the LBW group, we identified four core microbial communities: *Bacteroidales*, *Mogibacteriaceae*, *Parabacteroides*, and *Christensenellaceae*. In the HBW group, we identified seven core microbial communities: *Parabacteroides*, *Bacteroidales*, *Ruminococcaceae*, *Ruminococcus*, *Christensenellaceae*, *Paraprevotellaceae*, and *Bacteroides*. These results suggest that the gut microbiome of the HBW group of Yunong black pigs is more complex, with the core microbial genera not only functioning individually but also maximizing their regulation of the entire gut microbial network to jointly perform physiological functions. Similarly, we constructed the microbial networks of the HBMI and LBMI groups. The results showed that the HBMI group had two core microbial communities, *Bacteroides* and *Parabacteroides*, with a degree above 20. However, no core microbial communities with a degree above 20 were identified in the LBMI group. In the LBMI group, the highest core degree was observed in *Clostridiales* and *Bacteroidales*, both reaching a degree of 15. These results indicate that the gut microbiome structures of the HBW and HBMI groups are more stable, and they exhibit stronger nutritional absorption capabilities.

To further assess the distinct functions of different microecological environments and their potential impacts on the host, we conducted enrichment and differential analyses of the intestinal microbiota of Yunong pigs with varying growth performances using the KEGG database. The results of these analyses are depicted in Figure 3E,F. The intestinal microbiota of the HBW group exhibited significantly lower enrichment in functions such as the calcium signaling pathway, other types of O-glycan biosynthesis, the biosynthesis of type I polyketide backbone, and plant hormone signal transduction, compared to the LBW group (*p* < 0.05). However, it showed higher enrichment in functions like protein export, taurine and hypotaurine metabolism, D-glutamine and D-glutamate metabolism, and terpenoid backbone biosynthesis, although these differences were not statistically significant (*p* > 0.05). Similarly, the HBMI group’s intestinal microbiota significantly outperformed the LBMI group in functions related to nutrient absorption, including histidine metabolism, carbon fixation pathways in prokaryotes, protein processing in the endoplasmic reticulum, flagellar assembly, and oxidative phosphorylation. This suggests that the HBMI group’s gut microbiota possesses a stronger capacity for nutrient absorption compared to the LBMI group.

### 3.3. Identification of Microbiota Markers Significantly Associated with BW and BMI Through Multi-Model MWAS

Our preceding analyses have demonstrated a correlation between pig growth performance and the gut microbiota. The ensuing question is as follows: which components of the microbiota are responsible for this link, and can this influence be attributed to specific taxonomic units or to combinations of these units? To elucidate this, we performed an MWAS focusing on the microbial genera associated with the growth performance of Yunong black pigs. As depicted in Figure 3A, a Pearson correlation analysis identified 10 significant associations, while Wilcoxon rank-sum tests and an analysis of variance (ANOVA) detected seven and nine genera, respectively. A meta-analysis of various candidate microbes related to BW, selected by three models, pinpointed two key microbial communities—*Coprococcus* and *Blautia*—that exhibit a significant correlation with the BW phenotype in Yunong black pigs. Conversely, the MWAS analysis for BMI identified five associations through the Pearson correlation analysis, with eight and seven genera detected by Wilcoxon rank-sum tests and ANOVA, respectively. A comprehensive meta-analysis of the results from three models identified two microbial groups, *Ruminococcaceae* and *RF16*, that are significantly associated with BMI (Figure 3B).

Finally, to validate the relationship between the identified microbiota associated with the growth performance of Yunong black pigs and the phenotypes of BW and BMI, we conducted a trend analysis across the entire experimental cohort. The results, shown in Figure 3C–F, revealed that both *Coprococcus* and *Blautia* exhibited a significant negative correlation with BW, while *Ruminococcaceae* and *RF16* were significantly positively correlated with the BMI.

### 3.4. Association Between Host Genetics and the Key Microbiota Related to BMI and BW

To further investigate the relationship between the host and its microbiota composition, MGWASs were conducted using 35,933 SNPs genotyped in 461 animals alongside the abundance of four key microbial taxa. This analysis identified two genera that were significantly associated with variants: *Blautia* and *Coprococcus*. A total of four significant SNPs were distributed across two regions on the *Sus scrofa* chromosomes (SSCs): SSC4 and SSC8. Despite both genera belonging to the Lachnospiraceae family, no shared associated regions were identified for their abundances.

A total of three candidate genes, all classified as functional genes, were identified within or near the significant SNPs, as annotated in the *Sus scrofa* 11.1 genome assembly. Specifically, for the relative abundance of *Coprococcus*, three significant SNPs (CNC10081700, CNC10081722, and CNC10081827) on SSC8 are located near the functional candidate genes *INPP4B*, *SCOC*, and *PABPC4L*. In contrast, one significant SNP (CNC10042157) on SSC4, associated with the relative abundance of *Blautia*, does not correspond to any identified functional candidate gene.

**Figure 4:**
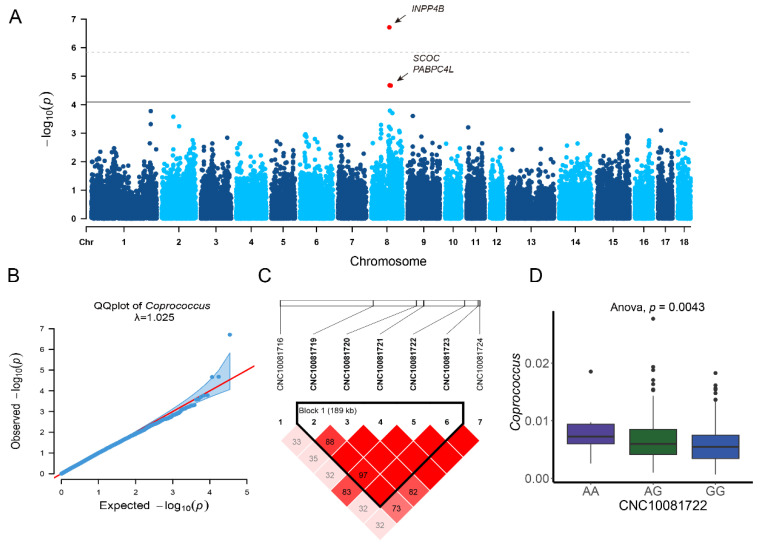
Manhattan plots and Q–Q plots of GWAS. (**A**) Manhattan plots of GWAS for the relative abundance of *Coprococcus*. (**B**) Q–Q plots showing the observed versus expected log *p* values for the relative abundance of *Coprococcus*; the estimated lambda (λ) is shown in the figure. (**C**) The LD blocks are marked with triangles. The values in the boxes are the LD (r^2^) between SNP pairs, and the boxes are colored according to the standard Haploview color scheme. (**D**) The effect of different genotypes with CNC10081722 on the abundance of *Coprococcus*.

A haplotype analysis revealed that the marker CNC10081722 is located within a long haplotype block spanning 189 kb in the *SCOC* gene (Figure 4A). Notably, significant differences in *Coprococcus* abundance were observed across the three genotypes of CNC10081722. The most common genotype, GG (*n* = 346), had an average *Coprococcus* abundance of 0.58%, followed by the AG genotype (*n* = 109) with a higher abundance of 0.69%. Although the AA genotype was the rarest (*n* = 6), it showed the highest *Coprococcus* abundance at 0.85% (Figure 4B,C).

Based on annotations from the *Sus scrofa* 11.1 genome assembly, functional genes located within or near the identified significant SNPs were identified. KEGG and GO analyses were subsequently performed to uncover the pathways and biological processes associated with the abundance of *Coprococcus* in the rectal microbiota of pigs. These analyses revealed that the functions related to inositol phosphate metabolism, phosphatidylinositol signaling, RNA binding, and the formation of ribonucleoprotein complexes and cytoplasmic stress granules are crucial for regulating metabolic pathways, gene expression, and cellular processes. These processes are essential for energy balance, fat storage, and growth, all of which are key factors influencing body weight.

## 4. Discussion

In recent years, the research on livestock growth performance has evolved beyond traditional phenotypic traits to explore the molecular and microbial factors that contribute to these variations. While host genetic variation is well established as a major determinant of phenotypic diversity, emerging studies emphasize the gut microbiome as an additional layer of complexity [20,21,22]. The gut microbiome has been associated with nutrient absorption [23,24,25], immune modulation, and pathogen suppression, highlighting its critical role in shaping growth-related traits [26,27]. However, the mechanisms by which host genetics influence microbiome composition and function remain poorly understood, particularly in pigs, where significant individual differences in growth performance persist even under uniform management conditions. To address this knowledge gap, our study takes an integrative approach that combines microbiome analyses with quantitative genetics. By applying microbial genome-wide association studies (MGWASs) in Yunong black pigs, we identified genetic factors influencing microbial communities and linked them to traits such as body weight (BW) and body mass index (BMI). This novel application of quantitative genetic tools in microbiome research not only enhances our understanding of host–microbiome interactions but also provides a framework for optimizing livestock production through precision microbiome management.

The gut microbiome, a complex ecosystem comprising thousands of bacteria, viruses, fungi, and protozoa, plays a crucial regulatory role in the host’s physiological functions such as nutrient absorption and growth development. In terms of nutrient absorption, studies have shown that 35% of the enzymes required for intestinal digestion are derived from microbes, with 25% of these active enzymes involved in carbohydrate metabolism [28]. Moreover, research has found that a high abundance of Escherichia in the ileum, identified as *Castellani, Chalmers* and *Brucella*, aids in the degradation of glucose and fructooligosaccharides [4,5]. In the cecum, *Actinomycete* promotes polysaccharide fermentation [6], while in the colon, *Bacterium* lactis and *Streptococcus* make significant contributions to lactate production [7]. In terms of metabolism, studies indicate that microbes can significantly impact host health, including conditions such as obesity and fat deposition [29]. Hildebrandt et al. demonstrated that the fat content in the diet is a cause of the imbalance in the gut microbiota of obese patients [30]. Other researchers have found that diseases such as intestinal inflammation, insulin resistance, type 2 diabetes, and hepatic steatosis are due to an increase in Gram-negative bacteria in the gut under high-fat diets, leading to elevated levels of metabolic endotoxins like lipopolysaccharides, which, in turn, damage the gastrointestinal barrier function [31,32,33]. In animal studies, Velagapudi et al. compared lipid metabolism in conventional mice with germ-free mice and found that conventional mice had increased levels of energy metabolites such as pyruvate, citrate, fumarate, and malate, while cholesterol and fatty acid levels were reduced. The increased rate of lipid removal suggests that gut microbes can participate in the host’s energy and lipid metabolism [34].

In this study, we first analyzed the diversity of the gut microbiome, enterotypes, and the differences in F/B ratios in Yunong black pigs with different growth performance phenotypes, and identified the core microbial communities that play major roles in their intestines. We found that the F/B ratio was significantly higher in the HBW group compared to the LBW group, and significantly lower in the HBMI group compared to the LBMI group. Studies have shown that an increased F/B ratio is often associated with obesity, which, to some extent, indicates a significant difference between the gut microbiota of the HWB group and the HBMI group. Although pigs in the HBW group have higher body weights, they may be obese, while HBMI indicates a more robust physique in pigs [35,36]. Subsequently, through a multiple model MWAS, we identified *Coprococcus*, *Blautia*, *Ruminococcaceae*, and *RF16* as significantly associated with pig growth performance. Notably, *Coprococcus* and *Blautia* showed a significant negative correlation with BW, while *Ruminococcaceae* and *RF16* exhibited a significant positive correlation with BMI. We hypothesize that these key microbial communities may play a crucial role in the host’s nutrient absorption and metabolism, thereby influencing the growth performance phenotypes of pigs. Research has found that *Coprococcus* is a probiotic with excellent therapeutic effects on colitis and can effectively alleviate diet-induced obesity in mice when fed through fermented food [37,38]. These findings, to some extent, explain the negative correlation between *Coprococcus* and BW. On the other hand, *Blautia* is also an anaerobic bacterium with probiotic characteristics. Studies have discovered that *Blautia*, when fed to mice on a high-fat diet, demonstrates the greatest ability to inhibit cellular lipid accumulation and effectively improve hyperlipidemia. The pathways through which *Blautia* inhibits hyperlipidemia have been elucidated through a combined analysis of microbiome, genomic, and pharmacological data [39]. These discoveries, to a certain extent, corroborate our research findings. *Ruminococcaceae* is the most abundant family of Firmicutes in the intestinal environment and plays a key role in the production of short-chain fatty acids. *Ruminococcaceae* is enriched in the gut microbiota during the later stages of pig growth and development, compared to the early stages [40]. Similarly, human studies have shown that *Ruminococcaceae* is more abundant in obese individuals than in lean individuals [41,42], which aligns with our findings. However, the direct causal relationship between *Ruminococcaceae*, body weight, and BMI remains unclear.

A further MGWAS analysis indicated that the key microbial communities, such as *Coprococcus* and *Blautia*, are not only associated with porcine BW or BMI but are also genetically regulated by the host. The *Blautia* community is regulated by host genetics. However, this significant locus was not annotated to any candidate gene. In this study, three candidate genes were identified as being associated with the abundance of *Coprococcus*. These findings provide insights into the genetic mechanisms regulating microbial populations and their potential influence on key traits such as growth performance in pigs. *Coprococcus*, a bacterium known for producing butyrate and propionate, is of particular interest as a potential indicator of health. Previous research in our lab identified *Coprococcus* as a key bacterium potentially influencing disease resistance in pigs. Its role in enhancing *SLA-DRB* gene expression was validated at the cellular level with increasing concentrations [43]. Previous studies have demonstrated a significant association between *Coprococcus* and growth traits, including body weight and backfat thickness, aligning with the findings of this study [44].

In this study, *PABPC4L* (Poly(A) Binding Protein Cytoplasmic 4 Like) is a protein-coding gene associated with *Coprococcus*. *PABPC4L* has been identified as being associated with atypical parkinsonian disorders [45]. Although research on *PABPC4L* is limited, it has been found to play a role in post-transcriptional RNA regulation, including RNA degradation, transport, binding, granulation, and the formation of ribonucleoprotein complexes. These functions are critical for regulating gene expression, maintaining metabolic homeostasis, and responding to cellular stress. Inositol Polyphosphate-4-phosphatase Type II B (*INPP4B*) was identified as being associated with the abundance of *Coprococcus*, a genus of gut microbiota as an enzyme in the phosphatidylinositol signaling pathway. Interestingly, additional significant loci for *INPP4B* were identified in lean mice [46]. Through functional annotation in KEGG and GO, it was revealed that *INPP4B* indirectly influences the balance of intestinal microbiota by regulating host metabolism, immunity, and the intestinal environment. This highlights the dynamic interplay between host signaling and microbiota, where changes in host functions can result in corresponding shifts in microbiota ecology, ultimately impacting host health. Phosphatidylinositol plays a key role in intramuscular fat deposition. Its supplementation enhances meat quality by regulating amino acid metabolism and modulating gut microbiota composition in fattening pigs. This, in turn, influences growth performance, apparent total digestibility, and the proliferation and function of intestinal epithelial cells [47,48,49]. The findings of this study suggest that *INPP4B* may influence BW through interactions between the gut microbiota and phosphatidylinositol.

The *SCOC* (Short Coiled-Coil Protein) is a protein-coding gene associated with *Coprococcus*. It functions as a novel positive regulator of starvation-induced autophagy, a highly conserved pathway essential for recycling cellular components, promoting stress survival, and maintaining cellular health and homeostasis [50]. Additionally, *SCOC* has been linked to immune cell infiltration levels [51]. Although the research on *SCOC* in pigs is limited, it has been annotated in GO as being associated with the trans-Golgi network (TGN). The TGN is a key component of Paneth cells, which are found in the small intestine of pigs [52]. This study found significant differences in bacterial abundance among the same genotypes of SNP loci linked to the *SCOC* gene, which may be related to the findings from the aforementioned research.

## 5. Conclusions

In summary, this study employed MWASs and a meta-analysis to identify the key microbial communities associated with pig growth performance. Additionally, MGWASs were utilized to uncover the genetic basis behind these microbial communities, thereby gaining a comprehensive understanding of their role in growth performance. Two microbial communities were found to be significantly correlated with genomic variations, greatly enriching the genetic information available for pig growth performance and laying the foundation for improving this trait in pigs. On the other hand, the study systematically elucidated the regulatory pathways between host genetics, gut microbiota, and host phenotypes using quantitative genetics methods. This provides theoretical and data support for future research on the interaction between host genetics and gut microbiota.

## Figures and Tables

**Table 1:** Composition and nutritional levels of the base diet.

Diet Composition	Content/%	Level of Nutrition	Content/%
Corn	63.10	Metabolizable energy/MJ·kg^−1^	14.23
Soybean meal (43%)	25	Crude protein	17.8
Bran	4	Total lysine	1.03
Choice white grease	2	Ca	0.71
Fish meal (67%)	2	Total phosphorus	0.56
Sow Vit-Min premix	0.50	Effective phosphorus	0.34
Salt	0.40		
Dicalcium phosphate	0.90		
Limestone	0.80		
Lysine	0.15		
Methionine	0.05		
Threonine	0.03		
Tryptophan	0.02		
Choline chloride	0.1		
Zeolite powder	0.95		
Total amount	100		

## Data Availability

The original contributions presented in this study are included in the article. Further inquiries can be directed to the corresponding authors.
